# Characteristics of Saliva in Syphilitics

**Published:** 1882-05

**Authors:** A. W. Harlan

**Affiliations:** Chicago.


### ARTICLE II.

### *Characteristics of Saliva in Syphilitics.*

BY DR. A. W. HARLAN, OF CHICAGO.

Not very much will be said concerning the saliva of persons infected with the virus of syphilis, for in most cases there is no appreciable difference existing between such saliva and that from the mouth of a perfectly healthy subject. It is not my purpose to dwell on the characteristics of syphilitic saliva, for the reason that they have no practical bearing in connection with the practice of dentistry, except as shall be mentioned further along; but the lesions of the mouth, resulting from inherited and acquired syphilis, are so numerous in form and variety, and are often destructive to the bones of the nose and mouth, that it is incumbent on all dental surgeons to be familiar with their appearance, duration, and treatment, primarily for the benefit of the patient, and secondarily for self protection. Therefore, it has been suggested that a brief resume of the order, character, and general appearance of mouth lesions may not prove uninteresting. Saliva from the mouth of a person suffering with syphilis in its second stage presents only this marked physical difference from saliva in general; a sufficient quantity collected for the purpose is poured into a vessel containing water; it sinks more quickly and does not mingle with the water to readily as non-syphilitic saliva; on testing, it is found to contain more albuminous matter, and when there are extensive patches and erosions of the mouth and pharynx, it is secreted in abundance, accompained by increase in size of the submaxillary glands; during the eruptive period of syphilis it is very much lessened in volume and gives an acid reaction. Saliva is the only one of the physiological secretions which is apt to be the vehicle in spreading the poison of syphilis, examples of which are here presented:—

In one case a sailor went from house to house tattooing, and was observed to wet the needles in his mouth before pricking in the colors. Fifteen out of twenty-two persons tattooed had acquired syphilis. On examination his mouth and throat were found covered with ulcers and erosions; the pus mingling with the saliva being pricked in with the coloring matter caused the inoculation. Two persons were indiscreet enough to use a tooth-brush which had previously been used by a syphilitic; they both acquired the disease. An old Frenchman smoked a pipe which had been used only a short time previously by a syphilitic friend, and in due course the initial chancre appeared on his lip. By searching the literature of syphilis, numerous cases may be found where kissing has been the means of communicating the disease. Two cases are reported in Woods Library of Medical Science, 1880, volume on venereal diseases, by E. L. Keyes, M. D. In twelve thousand cases reported from hospitals, three and one third per cent. exhibited the primary sore on the lip or within the mouth; more than ninety-four per cent. of the remainder showed the chancre on the genitals or anus. Whistles and trumpets sold on the streets and wet with the saliva of the venders, may be infectious. In some regions where glass-blowing establishments are numerous, laws have been enacted to prevent the use of the mouth-tubes, except by the owners, on account of the liability of communicating syphilis. Any article used by a syphilitic in the mouth when it is eroded or covered with ulcerous patches, may inoculate the innocent user, if it be not thoroughly cleansed. Many an innocent nurse has acquired syphilis by suckling a child inheriting it, as the mouth lesions appear so early in most cases that they are unable to escape unless the nipple remains unbraded. There are some who believe that the twisted end of a new cigar having been wet with saliva of a syphilitic may be contagious. The use of the rubber dam in the mouth of a syphilitic, and its subsequent use in the mouth of another, without perfect cleansing, might prove infectious; it would be if any blood were adherent, as it is known to be infectious. Forceps that have been used and not thoroughly cleansed; scalers and lancets, particularly abscess lancets, and other instruments used by dentists, oculists, and aurists, unless properly cleansed, would spread syphilis. (See Bumstead and Taylor, E. L. Keyes, and Ziemssen, on venereal diseases, for instances of infection by unclean instruments.) In circumcision and vaccination numerous cases are reported, especially in the latter. In transplanting teeth it would be possible to communicate syphilis, but J. Hunter's claim that because an abscess formed opposite the root of the transplanted tooth in three or four weeks after the operation, that it was a chancre, is of course erroneous; the abscess appeared because the pulp was not removed from the tooth, and in three or more weeks it decomposed and the gas had to have exit; hence the abscess. It is impossible to point out the numerous methods of acquiring syphilis in a brief paper. Suffice it to say that we cannot be too careful of our own fingers or our patients' mouth in any suspected case.

An abrasion or a surface capable of absorption, if it have, in contact with it, the poison of syphilis for a sufficient period of time, no matter where the spot be located, is as sure to exhibit the chancre as it is sure to result from sexual contact when one of the persons is diseased. From the fact that persons who acquire syphilis usually resort to the venereal specialist, dentists generally may be ignorant of the cause of the disease in its primary form, except from reading or when medically educated; but when the secondary symptoms progress far enough to exhibit excoriations, erosions, ulcers, and mucous patches in the mouth and throat, the dermatologist necessarily sends his patient to a dentist in order that all tartar shall be removed from the teeth, rough corners filed off and pieces of root removed, ill-fitting dentures trimmed and polished, so that the tongue, cheeks, and lips may be free from such sources of irritation. If the person infected falls into the hands of a dentist before consulting a physician, his knowledge of the clinical features of the disease ought to enable him to attend to the hygiene of the mouth so effectually that the treatment of the case constitutionally may be left to the venereal specialist until a cure is effected.

An outline may now be presented of the course of syphilis. After contact with the poison a chancre appears in three or four weeks, or longer; the chancre always appears at the point of inoculation; in two weeks thereafter the adjacent lymphatic glands enlarge, but seldom suppurate. After a month or six weeks has elapsed, an eruption on the skin, which may be quite general, appears, usually preceded by slight fever, accompained by headache, rheumatic pains at night, and an enlargement of the epi-trochlear and post-cervical glands; co-incident with these symptoms the mouth lesions are met with, consisting of mucous patches, erosions and ulcers; they are concomitant from time to time with other general symtoms of the disease during its whole course. After the expiration of a year, the mouth lesions are much worse, being usually whitened and excoriated patches, ragged ulcers upon the fauces and in the mouth, especially deep ulcers on the inside of the lower lip and opposite the last molar teeth. The tongue white and furred, which cannot be scraped off without bleeding, its edges and under surface raw, and the adjacent nasal mucous membrane covered with dark yellow and brownish scabs, a thickening and fissuring of the orifice of the nostrils, with the breath quite offensive. The mucous patch when once seen is not likely to be confounded with that from any other affection. It is nearly round and raised slightly, of a dirty white color, sometimes red or granulated, and covered with a puriform secretion; its size varies from a point to that of a large copper cent, patches occur on the tonsils, the whole of the pharynx, within the lips, the nose, trachea, larynx, and upon the tongue or under it. They are generally painless, unless surrounded by erythema, irritated by smoking, or a rough tooth, or some similar cause. A person with a mucous patch upon the lip or within it, is far more dangerous to a community than half a dozen people with developed chancres on the genitals, as may readily be seen when it is known that the secretion from it is infectious; lobbing of the tongue, which is often, observed, is generally the result of too vigorous use of mercury, and is not the result of syphilis in any stage. Chancre of the lip, which is most often seen in hospitals, is globular or orval, angry-looking, and the size of a nickel; many of you have seen it; it is highly infectious. Scaly patches appear in the mouth after two or more years, and their favorite locality is at the angle of the mouth, the tip, sides and dorsum of the tongue; frequently masses of overgrowth, with adherent epithelium, cover a patch under the tongue, which, when detached, are as thick as a knife blade; in some respects they resemble icthyosis, but a close inspection of this syphilide marks its color a mottled bluish white, and no mistake need be made concerning its origin; these usually require nothing more than local treatment. Gummy tumors of the hard and soft palate come without pain, but when once formed they destroy all tissue which has become infiltrated; they are peculiarly destructive of the bones of the mouth and nose, and need to be heroically treated with the iodides or mercury, internally.

It is generally agreed that syphilis causes the most extensive mouth and throat ravages, especially destructive of bone, when no previous scrofula or lupus can be traced; hence the disfigured nasal organs and injuries of the hard and soft palate, which are frequently irreparable. Gumma of the tongue differs from epithelioma in this: it occurs at any age, has syphilitic history, commences deep, and feels like a pea between the fingers, is some times multiple, and *may always be found on the posterior aspect on the sides, but never underneath the tongue.* When ulceration takes place, it uncovers a deep cavity, and is usually painless, which is characteristic. When excised completely, it does not recur. The appearance of mouth lesions from inherited syphilis is often as early as a week from birth to a few months or even years, yet the latter occurs very rarely. Snuffling, and a general coryza, with excoriations around the nose and mouth, indicate very plainly the cause of the trouble; the child whines, its voice is cracked, and the skin drawn tightly over the face, which gives it an oldish look, but the development of patches at the angles of the lids and orifice of the nostrils, are usual and correct points in diagnosis. After the seventh year the notched superior central incisors are a sure indication of inherited syphilis. The whole train of symptoms are observed from inherited, that are seen in acquired syphilis, except that there is no chancre: the lymphatic glands do not enlarge, and the nails are affected either by impairment of nutrition or ulcerative onychia, the latter of which is most frequently noticed.

### TREATMENT.

The local treatment of mouth lesions from acquired or inherited syphilis, is perfect cleanliness, the use of a soft brush and an alkaline powder or wash; the teeth should be polished and roots be extracted or pivoted; tobacco must be forbidden; and mild astringent washes and gargles be prescribed for young children. Strong warm tea with X grs. borax to the ℥ i, or V grs. chlorate of potash to the same, are effectual for infants and children. Patches and ulcers may be painted with a solution of nitrate of silver, or solution of corrosive chloride of mercury, grs. i i j to ℥ i alcohol, daily; or when necessary they should be touched lightly and carefully with a solution of acid nitrate of mercury twice a week, or any other favorite effectual remedy may be used. If there are gummata likely to involve the bone, iodide of potassium or iodide of sodium should be prescribed in V to X gr. doses or larger, during or after meals. The sodium causes less disturbance of the stomach. If the bones of the mouth have become denuded of their periosteum, no effort should be spared to prevent further caries or necrosis, but no local remedy will be found efficient. If the dentist be timid or wanting in definite knowledge of the needs of the patient, he should be sent to his physician without loss of time. In most cases the dead bone will separate from the living during prompt and appropriate constitutional treatment. Caries must be operated upon beyond the line of decay, then pursue a systemic treatment, which, if kept up sufficiently long, will certainly result in a cure.

It is hoped that if nothing new has been presented in this paper, it will serve to stimulate the unthinking to an investigation of the subject which has been so imperfectly sketched for your consideration.

### DISCUSSION.

Dr. Swain.—I have made a good many tests with a view to find whether the normal condition of the saliva is acid or alkaline, but without satisfactory results. I found the saliva in some cases acid, and in others alkaline, in persons of equally good health and similar habits, the tests being made from fifteen minutes to half an hour after eating. In more than half the cases it was acid. Twice during the year I have had in my office a harmlessly insane young lady. In her mouth I have found the saliva uniformly acid, but her teeth do not decay. She is about thirty years old, and has only three fillings in her teeth. Stringy saliva is almost always acid, and in such mouths, when the enamel turns white and begins to decay, the free use of preeipitated chalk will often check its progress.

Dr. Brophy.—Syphilis cannot be inherited from a father unless a mother has it. That is, the mother must acquire it before the child will inherit it. And it is not communicated from one person to another by the *physiological* secretions, but the saliva of syphilitic persons may often be mixed with more or less serum or pus from some abrasion on the lips or mouth.

Dr. Richards.—During the last few months I have made a great many tests of saliva, in all classes of mouths, both healthy and unhealthy. I found it alkaline in a majority of cases, and especially so in robust and healthy persons. I saw many cases of rapid decay in mouths having alkaline saliva, but between the teeth, where food was lodged and uncleanly, it was acid, and *there* was decay. If we can induce patients, after having had fillings made, to keep the proximal surfaces clean, the operations will usually be preserved. I believe a great deal of decay is caused by the food that lodges in the proximal spaces and remains till decomposed, for want of proper attention to cleanliness.

Dr. Black.—I have made many examinations of saliva with the same general results as described; but we never find decay without there being an acid condition *somewhere* in the mouth, and yet teeth do not *necessarily* decay because the saliva is acid, though they never decay without an acid. Many patients *always* present an acid saliva whose teeth have no decay. There is some other element in this question which we have not yet found out.

Dr. Spalding.—The reports indicate that the chemical condition of the saliva does not necessarily produce decay.* If properly developed and well calcified, teeth will resist all these conditions of the saliva, it is the duty of the dental profession to find out the cause of the defective development of human teeth now so prevalent.

Dr. Black.—I wish to say this. Imperfect development is not a cause of decay, but a condition favoring it. Decay is the destruction of the tooth, molecule by molecule, by some substance attacking it from without.

Dr. Swain.—I have arrived at the same conclusions as Dr. Black and Dr. Spalding, that the saliva is not very much responsible for decay. Unquestionably, the acids generated from food lodged at the necks of the teeth will produce decay.

Dr. Black.—The secretions of the mouth as they often become vitiated while contained there, I do regard as holding a causative relation to decay.

Dr. Spalding.—I agree with Dr. Black, except that in two cases of the most rapid decay that I have seen, I could find no acid reaction at any time, in very numerous tests, in all parts of the mouth. If the presence of an acid is essential to decay, how does decay begin in these cases? I have no doubt that after it has made some progress an acid reaction might be obtained within the cavities of decay. We have only just begun to investigate or to understand this subject.

Dr. Brophy.—Alkaline secretions will injure the cementum, and alkaline washes should be used with caution, if the cementum at the necks of the teeth is exposed.

---

*In this discussion the word "saliva" usually means the mixed fluids, saliva, mucus, etc., as found in the mouth.

Dr. Black exhibited photographs of a girl now thirteen years old, who at five years old lost the cheek and lips of one side by a slough caused by salivation. The lower jaw is firmly anchylosed. The development of the teeth has proceeded with this condition existing; the shedding of the temporary teeth and the growth of the permanent ones has been perfectly regular, except that the teeth stand a little outward from the normal position. The special point of interest in the case is this ; The buccal surfaces of all the exposes teeth have suffered from erosion, which is precisely the same in appearance as that which we find in normal mouths. The eroded surface is hard and smooth as glass, and some of the teeth are eroded almost to the pulp cavity. These teeth have never been used in chewing food ; they have not been bathed in the saliva, except as it overflows the mouth and runs out upon them, which it does a good deal. They have never been filed or cut in any way. As to this point I have the evidence of the girl herself, of her parents, and the family physician. The teeth of the opposite side have their proper covering of cheek and lips, and show no trace of erosion, and are otherwise perfectly normal. This case strikes me as furnishing a new fact in regard to abrasion or erosion of the teeth, or at least a case occurring under such novel conditions that I think it well to place it on record.—*Illinois State Dental Transactions.*

